# Tissue Reaction to Monofilament Grafts of Different Weights Used for Abdominal Wall Reinforcement

**DOI:** 10.3390/biomedicines14040896

**Published:** 2026-04-15

**Authors:** Milan Potić, Ivan Ignjatović, Dragoslav Bašić, Ljubomir Dinić, Bojan Vučković, Nebojša Jovanović, Slavica Stojnev

**Affiliations:** 1 Faculty of Medicine, University of Niš, 18000 Niš, Serbia; ignjatovic1964@gmail.com (I.I.); basicdr@gmail.com (D.B.); ljubomidinic@gmail.com (L.D.); slavicastojnev@gmail.com (S.S.); 2 Clinic for Urology, University Clinical Center Nis, 18000 Niš, Serbia; 3 Urology Department, General Hospital Prokuplje, 18400 Prokuplje, Serbia; drbojanvuckovic@gmail.com; 4 Center for Gynecology and Human Reproduction, Medical Military Academy, 11000 Belgrade, Serbia; nebojsa038@gmail.com; 5 Center for Pathology and Pathological Anatomy, University Clinical Center Nis, 18000 Niš, Serbia

**Keywords:** abdominal wall repair, rat, foreign body reaction, macrophages, fibroblast activation, immunohistochemistry

## Abstract

**Background/Objectives**: Hernia and pelvic organ prolapse often involve defects necessitating reinforcement with synthetic materials. Polypropylene meshes of varying weights are commonly used in abdominal wall repair; however, their impact on tissue response, potentially linked to adverse events during wound healing, remains incompletely understood. This study aimed to evaluate and compare the tissue response to lightweight polypropylene (LWPP) and heavyweight polypropylene (HWPP) grafts used for abdominal wall defect closure in Wistar rats. **Methods**: Abdominal wall defects were surgically created in male Wistar rats and repaired using either LWPP (43 g/m^2^) or HWPP (76 g/m^2^) mesh. Three weeks post-implantation, tissue response and wound closure were evaluated across different phases by assessing expression of CD68, CD56, GATA-3, CD138, vimentin, α-smooth muscle actin, and collagen deposition. **Results**: HWPP promoted a more pronounced foreign body response, as evidenced by increased CD68, CD56, and CD138 expression, while LWPP improved tissue compliance, enhanced fibroblast migration, and increased vimentin-associated cellular activity. **Conclusions**: Three weeks post-implantation, HWPP was found to induce a stronger inflammatory response, whereas LWPP implantation was associated with increased vimentin expression, indicating differences in the balance between inflammation and extracellular matrix remodelling during early wound healing.

## 1. Introduction

Hernia and pelvic organ prolapse are among the most common problems of the abdominal wall and are associated with fascial defects. The use of reinforcing material has been proven necessary to reinforce the abdominal wall during hernia surgery, as simple closure or endogenous tissue alone has been inadequate [1]. Recurrence rates exceed 50% for ventral hernias and 15% for groin hernias, indicating the significant challenges associated with hernia treatment without the reinforcement provided by foreign materials. Recurrence rates for abdominal hernias range from 17 to 30% [2]. The use of different materials, sutures, and mesh is part of everyday surgical interventions, and their utilization is, in most cases, unavoidable. Natural sutures are broken down by proteolysis, leading to a significant inflammatory response, whereas synthetic materials are hydrolysed, resulting in minimal tissue reaction. Since biocompatibility is an essential factor for both short- and long-term success in surgery and adequate wound closure, it is expected that suture material should be non-allergenic, non-carcinogenic, and produce minimal tissue reaction [3]. Nevertheless, the material used in everyday surgical practice has several disadvantages, including chronic pain, a foreign-body sensation, and seroma formation, all arising from the body’s reaction to foreign material [4].

During the wound-healing process, tissue response can be observed through different phases, namely inflammation, proliferation (granulation tissue and angiogenesis), and remodelling. In the initial phase, there is a dominance of inflammatory cells, granulocytes or agranulocytes, which can hamper wound healing. This phase is critical due to the elevated risk of infection and complications, which are seen very often. Approximately 60% of hospital infections are wound tissue infections associated with foreign materials, such as sutures, which facilitate bacterial adherence [5]. Macrophages play numerous roles in the wound healing process, influencing early inflammation, neutrophil clearance, and tissue remodelling [6]. Other inflammatory cells, such as T cells, contribute by reducing inflammation and promoting resolution and tissue repair via the Th2 response, while B cells (plasma cells) produce antibodies that modulate surrounding cells and tissues [7].

During the proliferation phase, in order to help tissue repair, fibroblasts, endothelial cells, and keratinocytes populate the tissue. Fibroblasts synthesize extracellular matrix components, e.g., collagen [8], and some fibroblasts differentiate into myofibroblasts, which are involved in wound contraction [9]. At the same time, due to angiogenesis, oxygen and nutrient supply are restored, helping tissue repair [10]. Keratinocytes at the wound edge migrate to the epithelialized surface and support other cells [10]. The interactions among these cells facilitate effective wound closure and progression to the remodelling phase. The remodelling phase begins at approximately 2–3 weeks and may persist for months. During this phase, collagen III is gradually replaced by collagen I, fibres reorganize along tension lines, and tissue tensile strength increases [8].

With the previously mentioned facts in mind, the aim of the current study is to evaluate tissue response to lightweight and heavyweight polypropylene grafts (LWPP and HWPP) used for abdominal wall defect closure in Wistar rats. This evaluation involves detailed microscopic examination of the inflammatory, proliferative, and scarring phases of wound healing. Expression of various molecular markers in immune and reactive cells will be analyzed and correlated with distinct wound-healing phases.

## 2. Materials and Methods

### 2.1. Material Analysis

In this study, low-weight polypropylene (LWPP; Gynecare Gynemesh, monofilament, Ethicon, Edinburgh, UK, 43 g/m^2^) and heavyweight polypropylene (HWPP; Prolene mesh, monofilament, Ethicon, Edinburgh, UK) were used. Scanning electron microscopy (SEM; JEOL JSM-5300, Tokyo, Japan) was employed to measure graft parameters, including pore size and filament thickness. Prior to analysis, native grafts were gold-coated for five minutes using the sputter-coating technique. SEM images were analyzed using ImageJ software (version 1.49v; Java, Oracle SE 7 (1.7) free software).

### 2.2. Animals and Housing

Male Wistar rats weighing 200–250 g were divided into two groups, with 12 animals per group. Prior to and during the experiment, animals were housed in group cages (6 per cage) under standard laboratory conditions with a temperature set at 22 ± 2 °C, a relative humidity of 50 ± 5%, and with a 12 h light/dark cycle. Food (standard chow) and water were provided ad libitum throughout the experiment. All procedures complied with the European Council Directive (EU Directive 2010/63/EU, 2021) and the Guide for the Care and Use of Laboratory Animals (10th edition, National Academy Press). The Ethics Committee for Animal Experimentation of the Faculty of Medicine, University of Nis, approved the study (No.01-2066-9, dated 1 April 2010). Experiments were conducted at the Biomedical Research Institute of the Faculty of Medicine, University of Nis.

### 2.3. Experimental Design

On the day of the experiment, animals were anesthetized with ketamine (10% Ketamidor; Richter, Wels, Austria) dosed according to body weight. Animals were placed supine, shaved, and a full-thickness abdominal wall defect measuring 20 × 25 mm was created. Using an overlay technique, 25 × 30 mm graft implants were applied for primary repair of the defect. In the first group, LWPP was used; in the second, HWPP grafts were used. After graft placement, an additional running suture without tension was applied. The skin and subcutis were closed with 3/0 polyglactin absorbable sutures (VicrylTM, Ethicon, Edinburgh, UK). Animals were sacrificed by ketamine overdose three weeks post-surgery.

### 2.4. Tissue Collection, Processing, and Immunohistochemical Staining

During necropsy, the entire abdominal wall was dissected (en bloc) with the graft centred, including at least 25 mm of adjacent tissue. Specimens were cut transversely to the animal’s long axis and fixed in 10% buffered formalin solution. After fixation, the tissue was dehydrated in increasing concentrations of ethanol (50%, 70%, 90%, and 100%). Subsequently, samples were embedded in paraffin blocks and sectioned serially into 3–5 µm slices. Tissue sections were stained using hematoxylin and eosin (HE) and Masson’s trichrome histochemical protocols.

For the immunohistochemical staining, tissue sections were incubated with following primary antibodies: CD68 (Abcam, Cambridge, UK, ab955, 1:100), CD56 (Abcam ab95153, 1:200), GATA3 (Abcam ab199428, 1:200), CD138 (Abcam ab130405, 1:100), and vimentin (Abcam ab8069, 1:1000). Briefly, tissue sections were deparaffinized in xylene and rehydrated in a graded series of ethanol and deionized water. After a heat-induced antigen retrieval procedure and peroxidase activity quenching, primary antibodies were applied and left overnight at 4 °C [11]. After incubation with appropriate HRP-conjugated secondary antibodies (Dako, Glostrup, Denmark), detection was performed with DAB, and slides were counterstained with hematoxylin, dehydrated, and mounted. Negative controls were included by omitting the primary antibody. A positive immunohistochemical reaction, the presence of the investigated protein, was verified as brown staining of cell membranes, cytoplasm, or cell nuclei by an experienced pathologist (SS).

### 2.5. Molecule Expression Quantification

Images of randomly selected visual fields (10 per slide) were captured at 200× magnification using an Olympus BX43 microscope (Olympus Corporation, Tokyo, Japan). Optical density (O.D.) analysis was performed using the ImageJ software (National Institutes of Health, Bethesda, MD, USA) as described previously [12,13,14]. Immunohistochemical analysis was performed using the ColourDeconvolution2 plugin (Version 2.1). H DAB was chosen from the top-down menu in the “ColorDeconvolution” plug-in to extract the DAB channel automatically for further analysis. In the toolbar, the “image” menu was opened, followed by “Adjust”, and the “Threshold” option was selected from the drop-down menu for the purpose of selecting the DAB-positive area. After that, the “create selection” option was used to mark the region of interest (ROI). The staining intensity was quantified by determining the mean grey value within the defined ROI. In ImageJ, a pixel’s value ranges from 0 to 255, where 0 is the darkest shade and 255 is the lightest shade. The obtained values were converted to optical density (OD) using the following formula: OD = (255/mean grey value).

### 2.6. Statistical Analysis

The results of the experiments were expressed as the mean ± SD. Statistically significant differences were determined by Student’s *t*-test for two small independent samples (GraphPad Prism version 5.03, San Diego, CA, USA). Probability values (*p*) less than or equal to 0.05 were considered to be statistically significant.

## 3. Results

### 3.1. Material Characteristics

**Figure 1:**
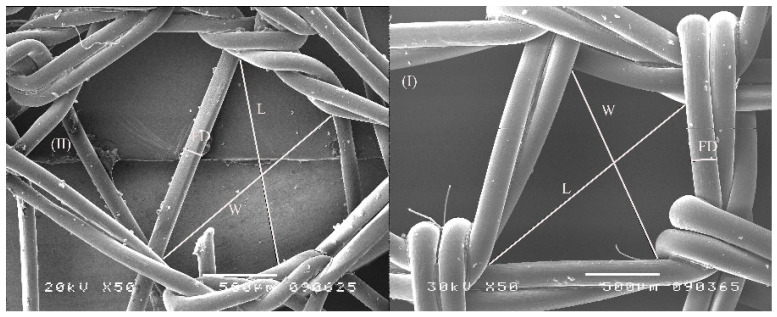
Electron microscopic images of material used in experiments with light (**left** (II)) and heavy (**right** (I)) weight graft material; L—length, W—width, FD—fiber diameter.

The measurement procedure and the results of the material analysis are given in Figure 1 and Table 1. SEM-focused characterization showed that LWPPs are built from significantly finer, thinner filaments and therefore exhibit significantly smaller effective pore spaces. On the other hand, HWPPs are built from thicker filaments and with higher areal density, yielding smaller interstices and greater overall thickness.

### 3.2. Tissue Appearance on Standard Histological Staining

**Figure 2:**
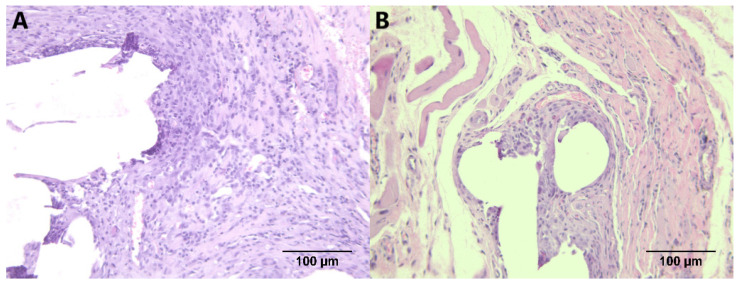
Microscopic appearance of tissue samples obtained from animals where light (**A**) and heavy (**B**) weight monofilament graft material was used; arrows indicate giant cells (×200 magnification).

The tissue of animals in which LWPP material was used appeared as newly formed granulation tissue, with occasional more mature tissue containing compact structures (Figure 2A). In animals with an HWPP graft, the tissue appeared as young granulation tissue, with increased numbers of fibroblasts, fibrocytes, and giant cells (Figure 2B).

### 3.3. Inflammatory Phase Related Markers Quantification

**Figure 3:**
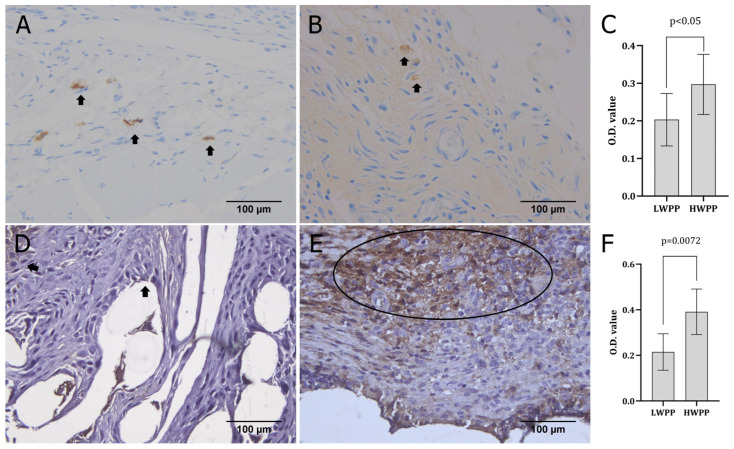
Expression patterns of CD68 and CD138 in tissue samples obtained from animals where LWPP (**A**,**D**) and HWPP (**B**,**E**) graft material was used. The expression was quantified (**C**,**F**). Arrows indicate CD68-positive cells (×400 magnification), while the circle shows the area of CD138-positive cells (×400 magnification).

The expression of CD68 and CD138 (Figure 3A,B,D,F) was quantified (Figure 3C,E). The CD68 expression pattern was randomly detected throughout the tissue sample in both cases (Figure 3A,B), and the quantification showed no significant difference in the staining intensity between the groups (Figure 3C). In the case of CD138 expression in rats where LWPP was placed, only random cells around the material appeared positive (Figure 3D), while in the case where HWPP was placed, a much larger area of positive cells was observed (Figure 3E). Comparing the quantified expression of CD138, it was found that this molecule is significantly more expressed in the group of animals where HWPP was used (Figure 3F).

### 3.4. Proliferative Phase Related Markers Quantification

**Figure 4:**
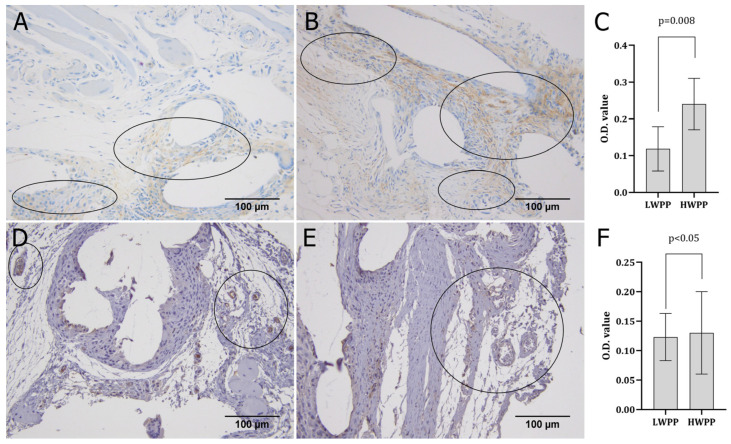
Immunohistochemical expression patterns of CD56 and GATA-3 in tissue samples obtained from animals where LWPP (**A**,**D**) and HWPP (**B**,**E**) graft material was used. The expression of both molecules was quantified (**C**,**F**). Expression patterns and localisation are circled (magnification ×200).

CD56 expression in both the LWPP and HWPP groups was observed in large, diffuse areas surrounding the material and throughout the newly formed tissue (Figure 4A,B), with greater dominance in rats where HWPP was used (Figure 4C). Immunohistochemical expression patterns of GATA-3 were sparse in both groups, with staining mainly in small groups of cells or around blood vessels (Figure 4D,E); the GATA-3 expression was shown to be almost identical in the examined groups (Figure 4F).

### 3.5. Remodelling Phase Related Markers Quantification

**Figure 5:**
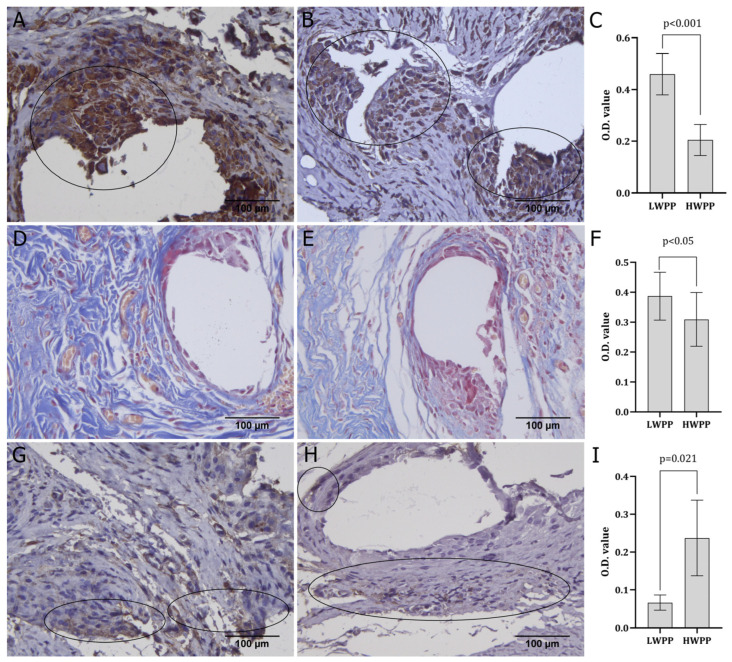
Immunohistochemical expression and histochemical patterns of vimentin, collagen, and actin in tissue samples obtained from animals where LWPP (**A**,**D**,**G**) and HWPP (**B**,**E**,**H**) graft material was used. The expression of examined molecules was quantified (**C**,**F**,**I**). Expression patterns and localisation are circled (magnification ×400).

Vimentin expression was observed in cells directly surrounding the mesh material in both the LWPP and HWPP groups (Figure 5A,B), with statistically higher expression in the LWPP group (Figure 5C). In both groups of animals, significant collagen deposition was observed in tissue sections stained with Masson’s trichrome (Figure 5D–F). The collagen fibres appeared pale blue with an irregular shape (Figure 5D,E), with no significant difference in the amounts between the groups (Figure 5F). The collagen fibres in the LWPP group were more irregular, whereas in the HWPP group, the fibres were more organized but fewer in quantity. Finally, the expression of actin molecules within the cell surrounding the mesh material was scarce in both the LWPP and HWPP groups (Figure 5G,H), with significant dominance in the HWPP group (Figure 5G,I).

## 4. Discussion

Material used during different operations aiming to fix the defects of the abdominal wall, e.g., places with reduced ability to sustain abdominal pressure, impacts the process of wound healing. It affects local tissue tension, tissue inflammatory response, fibroblast activation, and extracellular matrix deposition. A recent meta-analysis revealed that different weight meshes yield similar clinical outcomes, except that the HW mesh results in a more intense perception of a foreign body in operated patients [4]. Additionally, studies suggest that complication rates in vaginal surgery with HWPP are significant [15]. Some initial studies on rats, conducted back in the 1990s, that compared the tissue reactivity of various absorbable and non-absorbable, synthetic and natural, and braided and non-braided materials, found very little difference between the extent of tissue reaction [16]. However, more recent experimental studies using sophisticated methodology and a multidisciplinary approach did in fact show the difference in tissue reaction depending on the used material, as well as on the material features (e.g., flexibility, pore size, etc.) directly correlating with the vascular elements and fibroblast infiltration [16,17,18,19]. Based on a recent meta-analysis, the complication rate related to mesh placement in patients with vaginal hernia is 4.88% of all operations [20]. These complications are defined as those occurring shortly after surgical interventions, 30 days post-operation [20], which is partially encompassed in the study design. Although some complications related to mesh placement can be medium- and long-term, the mesh placement is somewhat debatable in prolapse surgery [20]. Apart from the direct complications from mesh placement, a recent cohort study indicates other factors that diminish life quality in these patients, which might be associated with mesh placement and operation options [21]. The resulting reaction of tissue 3 weeks after the surgery was the formation of granulation tissue, inflammatory response (Figure 2, Figure 3 and Figure 4), and the deposition of disorganized collagen fibres (Figure 2 and Figure 5). The observational study followed ISO 10993-6 standards in examining and interpreting the results [22].

Overlapping inflammatory and proliferative phases in the tissue samples were examined by measuring the expression of CD68, CD56, GATA-3, and CD138 (Figure 3 and Figure 4). In this phase, there are a few subphases that can be influenced by numerous factors and drive the tissue reaction in different directions [23]. During one of the subphases, there is a window during which adhesion to abdominal organs or other complications related to mesh placement (in the case of peritoneal/pelvic interventions) occurs [24], driven by CD68+ macrophages [23]. The expression of CD138 on different cells can be interpreted through different molecular pathways; however, in wound tissue, this molecule represents a relatively new biomarker of scar formation [25]. Thus, the molecules studied here specifically determine, to a certain extent, the type of cells in the remaining inflammatory response. Finally, the inflammatory cell profile is indicative of the state of wound healing [26] and can predict the outcome of the process [6,16]. Some previous studies suggested that a decrease in graft weight reduces the inflammatory foreign body reaction to a minimum [16], while others failed to produce such findings [23]. Similar conclusions to those from animal studies are drawn from clinical studies, indicating the need for further exploration in this field [27].

The role of CD68-positive cells (macrophages and histiocytes) is in the early phases of acute inflammation, during 48–72 h, and their number decreases as the time lapses [28]. These CD68-positive cells, dermal macrophages, have limited proliferative capabilities, although there are data indicating that they can potentially self-renew depending on the microenvironment or stage of wound healing [29]. In chronic inflammatory states, there is an increase in CD68 macrophages and a dysregulation between T and B cells, suggesting that these cells do have a role in other phases of wound healing as well [28]. The depletion of macrophages during the proliferative phase results in reduced collagen deposition and impaired wound closure [6]. This suggests that the signals arriving from macrophages are essential not only for the inflammatory phase but also for resolution and tissue regeneration [6]. The results of the present study indicate that the material of higher weight and thicker diameter enhances inflammatory tissue reaction with more intense dominance of CD68 macrophages than the material of lighter weight, but without a significant difference when compared to the group with LWPP material (Figure 3C).

The expression of CD138 in wound tissue can be brought into connection with the plasma cell infiltration. This type of reaction is associated with more chronic, antibody-mediated inflammatory responses seen in later stages of wound healing or in prolonged suture-associated foreign body reactions [29]. Additionally, it modulates cell activity and proliferation, especially that of keratinocytes [29]. In normal wound closure, the expression of this molecule in different cells is relatively low when compared to some other states (e.g., keloid) [24]. The present study found a significant difference in the CD138 expression (Figure 3F) in scar tissue of the rats in which grafts of different weights were applied, suggesting that the reaction intensity of CD138 cells is more pronounced in the HWPP group and that the potential differences in final scarring might be based on CD138 cell functioning.

CD56 is a cell surface glycoprotein frequently found on cells within the nervous system, bone marrow mesenchymal cells, and natural killer (NK) cells. In NK cells, CD56 expression is widely used to define distinct cell subsets with regulatory and cytotoxic functions [30,31,32,33]. Myofibroblasts, which are involved in muscle growth and regeneration, express CD56 and might reflect their progenitor status and regenerative potential [34]. Thus, CD56 can be interpreted as a molecule in both the inflammatory and proliferative phases of wound healing. In both samples with LWPP and HWPP, an increase in the number of CD56-positive cells (Figure 4A,B), with a significantly higher expression of this marker in rats with HWPP (Figure 4C), was observed. As mentioned, CD56 is a molecule that can also be found in other cells as well (e.g., myofibroblasts), potentially indicating muscle regeneration at the wound edges or even nerve ingrowth that could have occurred during the 3-week study period.

The examined molecule, GATA-3, is a transcription factor that regulates gene expression in various cells, primarily influencing reparative immune cells during the inflammatory and proliferative phases of wound healing [35]. Its immunoregulatory role during inflammation involves polarization and promotion of type-2 immune responses through the suppression of pro-inflammatory cytokines and upregulation of modulatory cytokines [36]. During early inflammation, GATA-3 and CD56-positive innate lymphoid and NK cells participate in immune regulation and cytokine signalling, facilitating inflammation resolution and transition to the proliferative phase [35]. GATA-3 expression in newly formed tissue was not different between the HWPP and LWPP groups (Figure 4F).

The final wound healing phase involves tissue remodelling, characterized by decreased cellularity, contraction, and scar maturation. Various molecules and cells mediate tissue changes and adaptation to form mature connective tissue. Vimentin production is associated with macrophage activity, partially observed via CD68 staining in this study (Figure 3A–C), and is also present in fibroblasts [37]. Extracellular vimentin can act as a damage-associated molecular pattern (DAMP), potentially exacerbating inflammatory responses [38]. Additionally, vimentin regulates fibroblast proliferation, myofibroblast transition [39], and collagen production and deposition [40]. Although vimentin production was higher in the LWPP group than in the HWPP group (Figure 5C), this cannot be solely attributed to CD68-positive macrophages, which were almost equally abundant in the groups. Instead, vimentin levels in the LWPP group may correlate with collagen amounts and α-smooth muscle actin expression (Figure 5).

During the final wound closure phase, myofibroblasts expressing α-smooth muscle actin contribute to wound contraction [9] and collagen secretion for stabilization [4]. However, excessive mesh-induced reactions can provoke myofibroblast formation and tissue fibrosis, leading to pain, a common postoperative complication [41]. The described complications frequently follow mesh placement surgery and are typically described as medium and long-term [20], requiring different times to occur. This may result in altered collagen deposition and reduction, increased cell apoptosis, proteolysis, and mesh displacement, causing various complications. In this study, both LWPP and HWPP induced limited α-smooth muscle actin expression in examined tissue samples, with more intense expression in HWPP (Figure 5G–I). This indicates that HWPP might have impacted the myofibroblast more intensely than LWPP. Variable tissue reactions and myofibroblast proliferation have been reported with other mesh types, such as ultra-lightweight mesh, suggesting that implantation position and pore size may influence postoperative complications [41].

Collagen organization is crucial for wound reinforcement, with deposition closely linked to myofibroblast function [4]. Six weeks after different mesh placements, the collagen deposition amount and fibre orientation did not significantly differ by material type. The only notable difference in collagen quantity was observed between titanium-coated and multifilament polypropylene meshes, correlating with tissue oxidative stress levels [16]. In this study, significantly greater collagen deposition was observed in rats implanted with HWPP compared to those with LWPP (Figure 5D,F). Collagen fibre quantity at three weeks post-surgery suggests progression into the final wound closure phase. This may indicate that wound closure in the HWPP group advanced more rapidly than in the LWPP group. Also, collagen fibre thickness and organization were poorer in the LWPP group compared to the HWPP group.

Based on the present study’s findings, the difference between the studied mesh materials can be attributed to their intrinsic structural and physicochemical properties, particularly filament thickness, mesh density, and effective pore size. HWPP promotes a more pronounced foreign body response, as evidenced by increased CD68, CD56, and CD138 expression. On the other hand, LWPP improved tissue compliance, enhanced fibroblast migration, and increased vimentin-associated cellular activity, indicating a more favourable microenvironment for early remodelling despite less organized collagen deposition at this stage. The results of the present study indicate that mesh type significantly influences the balance between inflammatory and regenerative processes, with LWPP being more favourable to functional tissue integration, while HWPP is more favourable for generating more stability with residual inflammatory response.

## 5. Conclusions

The results indicate that mesh weight affects tissue reaction three weeks post-implantation. Animals implanted with HWPP mesh exhibited a more pronounced inflammatory response, as evidenced by increased expression of CD138, CD56, and GATA-3. On the other hand, animals with LWPP mesh showed higher vimentin expression and significantly greater collagen deposition. These findings suggest that mesh weight modulates the balance between inflammatory response, extracellular matrix deposition, and reorganization during early wound closure. Future studies should investigate longer-term effects of LWPP and HWPP to elucidate differences in inflammatory responses, collagen deposition and reorganization, scar maturation, and tissue integration.

## Figures and Tables

**Table 1:** Basic characteristics of the used grafts.

Material Type	Weight (g/m^2^)	Pore Size (mm^2^)	*p*-Value	Fibre Thickness (mm)	*p*-Value
LWPP	43	0.49 ± 0.01	>0.01	0.068 ± 0.009	>0.01
HWPP	76	0.57 ± 0.02	>0.01	0.085 ± 0.013	>0.01

## Data Availability

Data will be given upon request from the corresponding author.

## Abbreviations

The following abbreviations are used in this manuscript:

CD68	Cluster of Differentiation 68
CD56	Cluster of Differentiation 56 (Neural Cell Adhesion Molecule, NCAM)
CD138	Cluster of Differentiation 138 (Syndecan-1)
GATA-3	GATA Binding Protein 3
LWPP	Lightweight Polypropylene Mesh
HWPP	Heavyweight Polypropylene Mesh
SEM	Scanning Electron Microscopy
HE	Hematoxylin and Eosin
HRP	Horseradish Peroxidase
DAB	3,3′-Diaminobenzidine
ROI	Region of Interest
O.D.	Optical Density
SD	Standard Deviation
STAT3	Signal Transducer and Activator of Transcription 3
ILC2	Type 2 Innate Lymphoid Cells
TLR4	Toll-Like Receptor 4
TGF-β	Transforming Growth Factor Beta
